# 2-Chloro-5-chloro­meth­yl-1,3-thia­zole

**DOI:** 10.1107/S1600536811019052

**Published:** 2011-05-25

**Authors:** Ling-Ling Zhao, Wei-Hua Cheng, Zhao-Sheng Cai

**Affiliations:** a Department of Pharmacy Engineering, College of Chemical and Biological Engineering, Yancheng Institute of Technology, Yancheng 224051, People’s Republic of China; b Department of Chemical Engineering, Yancheng College of Textile Technology, Yancheng 224051, People’s Republic of China

## Abstract

In the title compound, C_4_H_3_Cl_2_NS, the chloro­methyl C and 2-position Cl atoms lie close to the mean plane of the thia­zole ring [deviations = 0.0568 (2) and 0.0092 (1) Å, respectively]. No classical hydrogen bonds are found in the crystal structure.

## Related literature

The title compound is an inter­mediate in the manufacture of agrochemicals, see: Kozo *et al.* (1986 ▶). For the synthesis of the title compound, see: Beck & Heitzer (1988 ▶); For bond-length data, see: Allen *et al.* (1987 ▶).
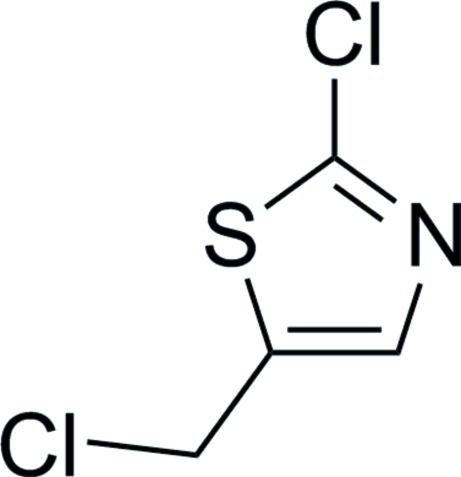


## Experimental

### 

#### Crystal data

C_4_H_3_Cl_2_NS
*M* *_r_* = 168.03
Monoclinic, 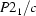

*a* = 4.2430 (8) Å
*b* = 17.151 (3) Å
*c* = 9.1640 (18) Å
β = 96.82 (3)°
*V* = 662.2 (2) Å^3^
*Z* = 4
Mo *K*α radiation
μ = 1.18 mm^−1^
*T* = 293 K
0.30 × 0.20 × 0.10 mm

#### Data collection

Enraf–Nonius CAD-4 diffractometer
Absorption correction: ψ scan (North *et al.*, 1968 ▶) *T*_min_ = 0.718, *T*_max_ = 0.891
2697 measured reflections
1211 independent reflections
932 reflections with *I* > 2σ(*I*)
*R*_int_ = 0.060
3 standard reflections every 200 reflections  intensity decay: 1%

#### Refinement

*R*[*F* ^2^ > 2σ(*F* ^2^)] = 0.043
*wR*(*F* ^2^) = 0.151
*S* = 1.00
1211 reflections
74 parameters
H-atom parameters constrained
Δρ_max_ = 0.30 e Å^−3^
Δρ_min_ = −0.28 e Å^−3^

### 

Data collection: *CAD-4 Software* (Enraf–Nonius, 1985 ▶); cell refinement: *CAD-4 Software*; data reduction: *XCAD4* (Harms & Wocadlo, 1995 ▶); program(s) used to solve structure: *SHELXS97* (Sheldrick, 2008 ▶); program(s) used to refine structure: *SHELXL97* (Sheldrick, 2008 ▶); molecular graphics: *SHELXTL* (Sheldrick, 2008 ▶); software used to prepare material for publication: *SHELXTL*.

## Supplementary Material

Crystal structure: contains datablocks I, global. DOI: 10.1107/S1600536811019052/vm2096sup1.cif

Structure factors: contains datablocks I. DOI: 10.1107/S1600536811019052/vm2096Isup2.hkl

Supplementary material file. DOI: 10.1107/S1600536811019052/vm2096Isup3.cml

Additional supplementary materials:  crystallographic information; 3D view; checkCIF report

## Figures and Tables

**Fig. 1.**
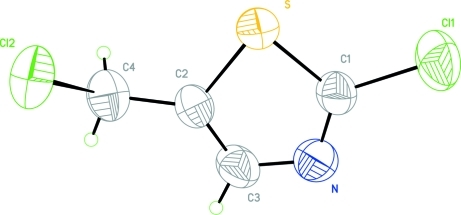
The molecular structure of (I), with the atom-numbering scheme. Displacement ellipsoids are drawn at the 50% probability level.

## supplementary crystallographic information

### Comment

The title compound, 2-chloro-5-(chloromethyl)thiazole is an important intermediate for manufacturing agrochemicals (Kozo *et al.*, 1986).

The molecular structure of (I) is shown in Fig. 1. The bond lengths and angles are within normal ranges (Allen *et al.*, 1987).

The thiazole ring is planar (max. deviation of 0.000 (5) Å  for C3). Atoms C4 and Cl1 lie close to this mean plane, whereas atom Cl2 is 1.4090 (1) Å out of the thiazole plane. The torsion angle S—C2—C4—Cl2 is -66.66 (1) °. The shortest distance between the centroids of the thiazole rings in the packing is 5.554 (1) Å.

### Experimental

The title compound, (I) was prepared by the method of chlorination-cyclization reaction reported in literature (Beck & Heitzer, 1988). The crystals were obtained by dissolving (I) (0.2 g, 1.2 mmol) in ethanol (25 ml) and evaporating the solvent slowly at room temperature for about 5 d.

### Refinement

H atoms were positioned geometrically (C—H = 0.93–0.97 Å) and refined using a riding model with *U*_iso_(H) = 1.2*U*_eq_(C).

### Crystal data

**Table:**

C_4_H_3_Cl_2_NS	*F*(000) = 336
*M**_r_* = 168.03	*D*_x_ = 1.686 Mg m^−^^3^
Monoclinic, *P*2_1_/*c*	Mo *K*α radiation, λ = 0.71073 Å
Hall symbol: -P 2ybc	Cell parameters from 25 reflections
*a* = 4.2430 (8) Å	θ = 10–13°
*b* = 17.151 (3) Å	µ = 1.18 mm^−^^1^
*c* = 9.1640 (18) Å	*T* = 293 K
β = 96.82 (3)°	Block, colourless
*V* = 662.2 (2) Å^3^	0.30 × 0.20 × 0.10 mm
*Z* = 4	

### Data collection

**Table:**

Enraf–Nonius CAD-4 diffractometer	932 reflections with *I* > 2σ(*I*)
Radiation source: fine-focus sealed tube	*R*_int_ = 0.060
graphite	θ_max_ = 25.4°, θ_min_ = 2.4°
ω/2θ scans	*h* = 0→5
Absorption correction: ψ scan (North *et al.*, 1968)	*k* = −20→20
*T*_min_ = 0.718, *T*_max_ = 0.891	*l* = −11→10
2697 measured reflections	3 standard reflections every 200 reflections
1211 independent reflections	intensity decay: 1%

### Refinement

**Table:**

Refinement on *F*^2^	Secondary atom site location: difference Fourier map
Least-squares matrix: full	Hydrogen site location: inferred from neighbouring sites
*R*[*F*^2^ > 2σ(*F*^2^)] = 0.043	H-atom parameters constrained
*wR*(*F*^2^) = 0.151	*w* = 1/[σ^2^(*F*_o_^2^) + (0.098*P*)^2^] where *P* = (*F*_o_^2^ + 2*F*_c_^2^)/3
*S* = 1.00	(Δ/σ)_max_ < 0.001
1211 reflections	Δρ_max_ = 0.30 e Å^−^^3^
74 parameters	Δρ_min_ = −0.28 e Å^−^^3^
0 restraints	Extinction correction: *SHELXS97* (Sheldrick, 2008), Fc^*^=kFc[1+0.001xFc^2^λ^3^/sin(2θ)]^-1/4^
Primary atom site location: structure-invariant direct methods	Extinction coefficient: 0.030 (8)

### Special details

**Table:**

Geometry. All e.s.d.'s (except the e.s.d. in the dihedral angle between two l.s. planes) are estimated using the full covariance matrix. The cell e.s.d.'s are taken into account individually in the estimation of e.s.d.'s in distances, angles and torsion angles; correlations between e.s.d.'s in cell parameters are only used when they are defined by crystal symmetry. An approximate (isotropic) treatment of cell e.s.d.'s is used for estimating e.s.d.'s involving l.s. planes.
Refinement. Refinement of *F*^2^ against ALL reflections. The weighted *R*-factor *wR* and goodness of fit *S* are based on *F*^2^, conventional *R*-factors *R* are based on *F*, with *F* set to zero for negative *F*^2^. The threshold expression of *F*^2^ > σ(*F*^2^) is used only for calculating *R*-factors(gt) *etc*. and is not relevant to the choice of reflections for refinement. *R*-factors based on *F*^2^ are statistically about twice as large as those based on *F*, and *R*- factors based on ALL data will be even larger.

### Fractional atomic coordinates and isotropic or equivalent isotropic displacement parameters (Å^2^)

**Table:**

	*x*	*y*	*z*	*U*_iso_*/*U*_eq_	
S	0.3422 (2)	0.57491 (5)	0.76517 (10)	0.0589 (4)	
N	0.5235 (10)	0.71113 (19)	0.8427 (3)	0.0725 (10)	
Cl1	0.7424 (3)	0.60986 (7)	1.04471 (11)	0.0811 (5)	
C1	0.5381 (9)	0.6400 (2)	0.8830 (3)	0.0530 (9)	
Cl2	0.2025 (2)	0.58243 (7)	0.38099 (10)	0.0671 (4)	
C2	0.2262 (8)	0.6502 (2)	0.6483 (3)	0.0484 (8)	
C3	0.3450 (12)	0.7162 (2)	0.7087 (4)	0.0684 (11)	
H3A	0.3078	0.7639	0.6615	0.082*	
C4	0.0159 (10)	0.6382 (2)	0.5089 (4)	0.0637 (10)	
H4A	−0.0443	0.6886	0.4660	0.076*	
H4B	−0.1761	0.6119	0.5297	0.076*	

### Atomic displacement parameters (Å^2^)

**Table:**

	*U*^11^	*U*^22^	*U*^33^	*U*^12^	*U*^13^	*U*^23^
S	0.0750 (7)	0.0467 (5)	0.0523 (6)	−0.0053 (4)	−0.0033 (4)	0.0014 (4)
N	0.109 (3)	0.0552 (18)	0.0516 (18)	−0.016 (2)	0.0015 (18)	−0.0070 (14)
Cl1	0.0967 (9)	0.0963 (9)	0.0464 (6)	0.0075 (6)	−0.0078 (5)	0.0011 (5)
C1	0.065 (2)	0.055 (2)	0.0386 (17)	−0.0016 (17)	0.0064 (15)	−0.0013 (14)
Cl2	0.0682 (7)	0.0810 (7)	0.0494 (6)	−0.0024 (5)	−0.0041 (4)	−0.0146 (4)
C2	0.0480 (19)	0.0554 (19)	0.0427 (17)	0.0070 (15)	0.0090 (14)	0.0017 (14)
C3	0.102 (3)	0.0468 (19)	0.055 (2)	0.003 (2)	0.005 (2)	0.0037 (17)
C4	0.057 (2)	0.076 (2)	0.057 (2)	0.0128 (19)	0.0042 (18)	−0.0003 (19)

### Geometric parameters (Å, °)

**Table:**

S—C1	1.700 (4)	C2—C3	1.333 (5)
S—C2	1.712 (3)	C2—C4	1.482 (5)
N—C1	1.275 (5)	C3—H3A	0.9300
N—C3	1.367 (5)	C4—H4A	0.9700
Cl1—C1	1.705 (3)	C4—H4B	0.9700
Cl2—C4	1.772 (4)		
			
C1—S—C2	89.16 (17)	C2—C3—H3A	121.3
C1—N—C3	108.9 (3)	N—C3—H3A	121.3
N—C1—S	116.2 (3)	C2—C4—Cl2	112.0 (3)
N—C1—Cl1	122.9 (3)	C2—C4—H4A	109.2
S—C1—Cl1	120.9 (2)	Cl2—C4—H4A	109.2
C3—C2—C4	129.4 (3)	C2—C4—H4B	109.2
C3—C2—S	108.3 (3)	Cl2—C4—H4B	109.2
C4—C2—S	122.3 (3)	H4A—C4—H4B	107.9
C2—C3—N	117.5 (3)		
			
C3—N—C1—S	0.0 (5)	C4—C2—C3—N	177.2 (4)
C3—N—C1—Cl1	179.6 (3)	S—C2—C3—N	0.0 (5)
C2—S—C1—N	0.0 (4)	C1—N—C3—C2	0.0 (6)
C2—S—C1—Cl1	−179.6 (2)	C3—C2—C4—Cl2	116.5 (4)
C1—S—C2—C3	0.0 (3)	S—C2—C4—Cl2	−66.6 (4)
C1—S—C2—C4	−177.4 (3)		

## References

[bb1] Allen, F. H., Kennard, O., Watson, D. G., Brammer, L., Orpen, A. G. & Taylor, R. (1987). *J. Chem. Soc. Perkin Trans. 2*, pp. S1–19.

[bb2] Beck, G. & Heitzer, H. (1988). US Patent No. 4748243.

[bb3] Enraf–Nonius (1985). *CAD-4 Software* Enraf–Nonius, Delft, The Netherlands.

[bb4] Harms, K. & Wocadlo, S. (1995). *XCAD4* University of Marburg, Germany.

[bb5] Kozo, S., Shinichi, T., Shinzo, K. & Koichi, M. (1986). EP Patent No. 0192060.

[bb6] North, A. C. T., Phillips, D. C. & Mathews, F. S. (1968). *Acta Cryst.* A**24**, 351–359.

[bb7] Sheldrick, G. M. (2008). *Acta Cryst.* A**64**, 112–122. 10.1107/S0108767307043930 18156677

